# Supplementary material for: Alarm communication predates eusociality in termites
Source: Commun Biol. 2023 Jan 21;6:83. doi: 10.1038/s42003-023-04438-5 (PMC9867704; doi:10.1038/s42003-023-04438-5)
Supplement: Supplementary file 2 — Supplementary Information [file 42003_2023_4438_MOESM2_ESM.pdf]

Supplementary Information for

Alarm communication predates eusociality in termites

David Sillam-Dussès, Vojtěch Jandák, Petr Stiblik, Olivier Delattre, Thomas Chouvenc, Ondřej Balvín, Josef Cvačka, Delphine Soulet, Jiří Synek, Marek Brothánek, Ondřej Jiříček, Michael S. Engel*, Thomas Bourguignon, Jan Šobotník*

* Michael S. Engel (msengel@ku.edu) & Jan Šobotník (sobotnik@ftz.czu.cz)

Included in this document:

- Figures S1-S6
- Tables S1-S4
- References not included in the main text

Fig. S1: GC/MS full-scan chromatograms of compounds from soldier heads obtained by hexane extraction (A, C) or from SPME-headspace (B, D) in *Mastotermes darwiniensis* (A, B) and *Glossotermes oculatus* (C, D).

Mastotermes darwiniensis: **a**: Heads of 5 soldiers were extracted in 32 µl of hexane, and 1 µl of the extract was injected into the GC/MS. Data show a strong signal of *p*-benzoquinone and numerous peaks of hydrocarbons, likely of cuticular origin. **b**: Heads of 5 soldiers were crushed at the bottom of a 2-mL glass vial. The vial was sealed, the SPME holder needle was passed through the vial septum, and the fiber was exposed for 10 minutes at room temperature. The volatiles were desorbed in the GC injector. Data show a strong signal of *p*-benzoquinone and three siloxane peaks marked by an asterisk (laboratory contamination).

Glossotermes oculatus: **c**: Heads of 10 soldiers were extracted as described above for *M. darwiniensis*. Data show the absence of any specific compound which plays the role of the alarm pheromone. The peaks at higher retention times (Chol) can be attributed to aliphatic compounds with high molecular weight, likely hydrocarbons (tR = 28.5 min and 30.4 min) or cholesterol (tR = 26.9 min). **d**: Heads of 6 soldiers were crushed and analyzed by SPME as described above for *M. darwiniensis*. Data show the absence of any specific compound which could be the alarm pheromone in this species.

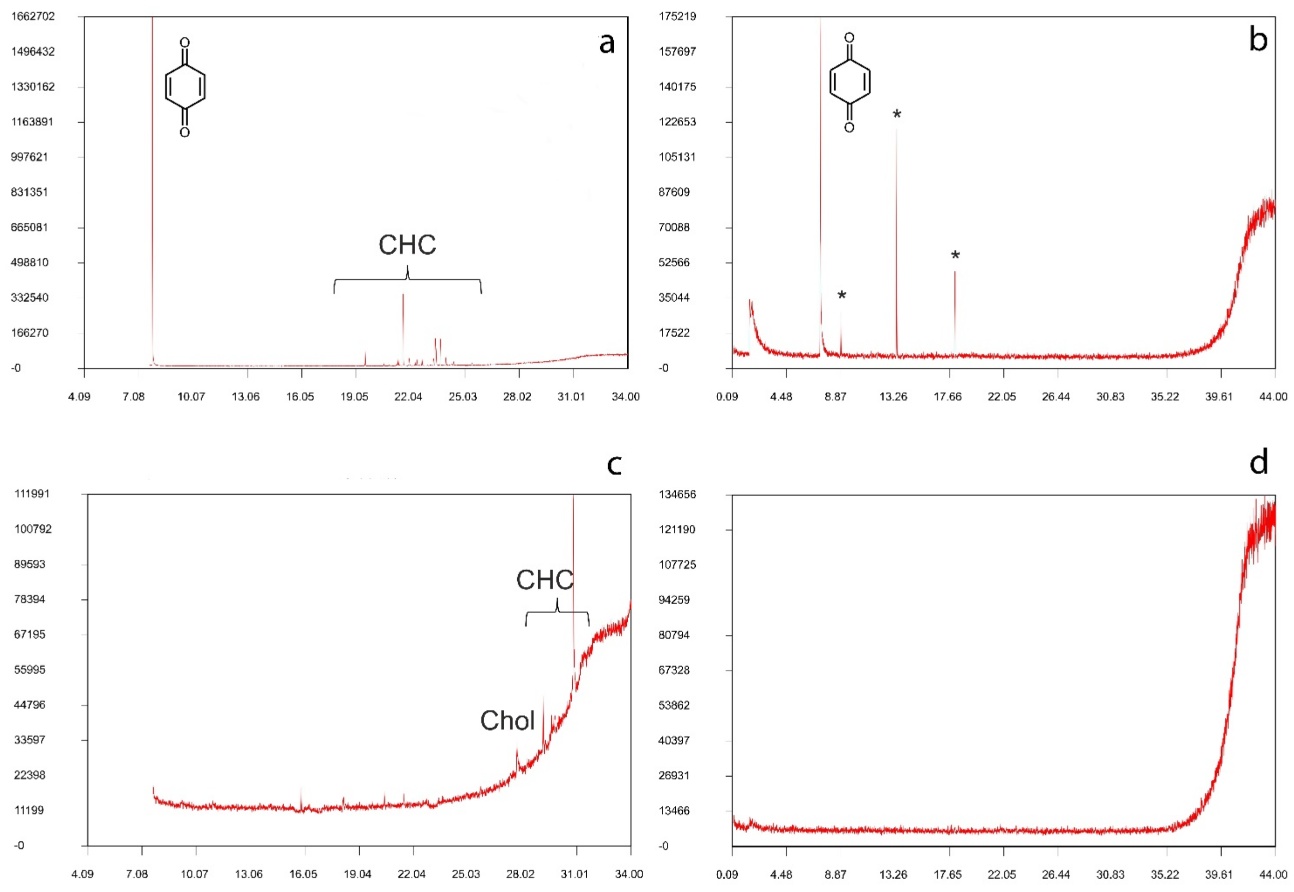

Fig. S2: Correlation analysis between ecological characters and alarm characters. Correlation is indicated by green fields; blank means no correlation.

	Body size ratio between castes	Worker subcastes	Soldier subcastes	Life type
**Drumming in Workers**	☺		☺	
**Tremulation in Soldiers**	☺	☺		
**Chemical alarm presence**				☺

Fig. S3: Schematic representation of the vibroacoustic alarm communication. Black upward arrows represent tremulation beats, blue upward arrows represent drumming beats, and blue downward arrows represent head-banging beats. The beats are separated by short (at high frequency; in green) or long (at low frequency; in yellow) breaks_and the average duration between each beat is indicated.

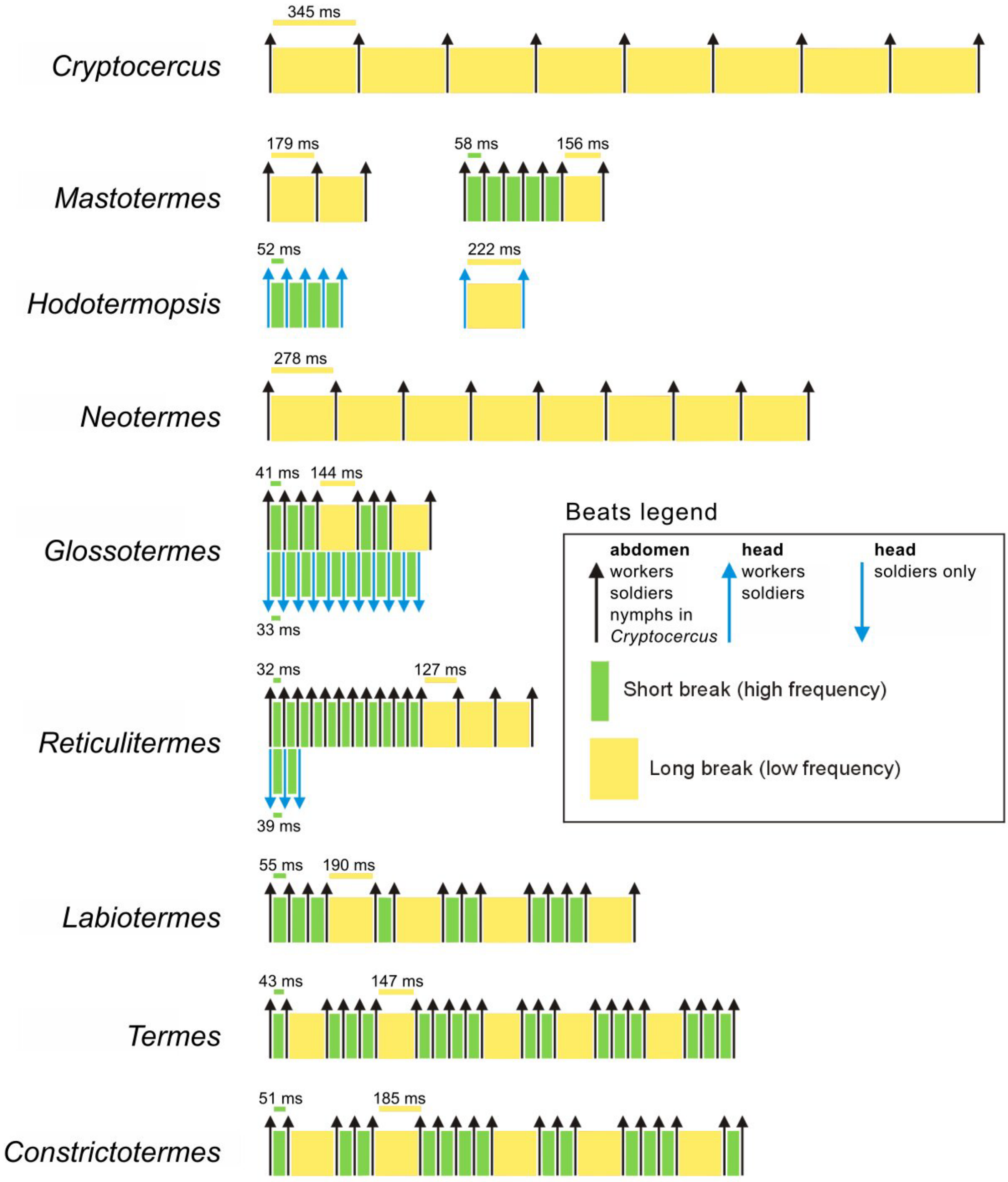

Fig. S4: Stability of vibrations frequency. Stability of pauses in high tremulations (a) and head-banging (b) among studied termites. The values are given as percentual difference from the mean duration. On each box, the central mark is the median, the edges of the box are the 25th and 75th percentiles, the whiskers extend to the most extreme datapoints the algorithm considers to be not outliers, and the outliers are plotted individually.

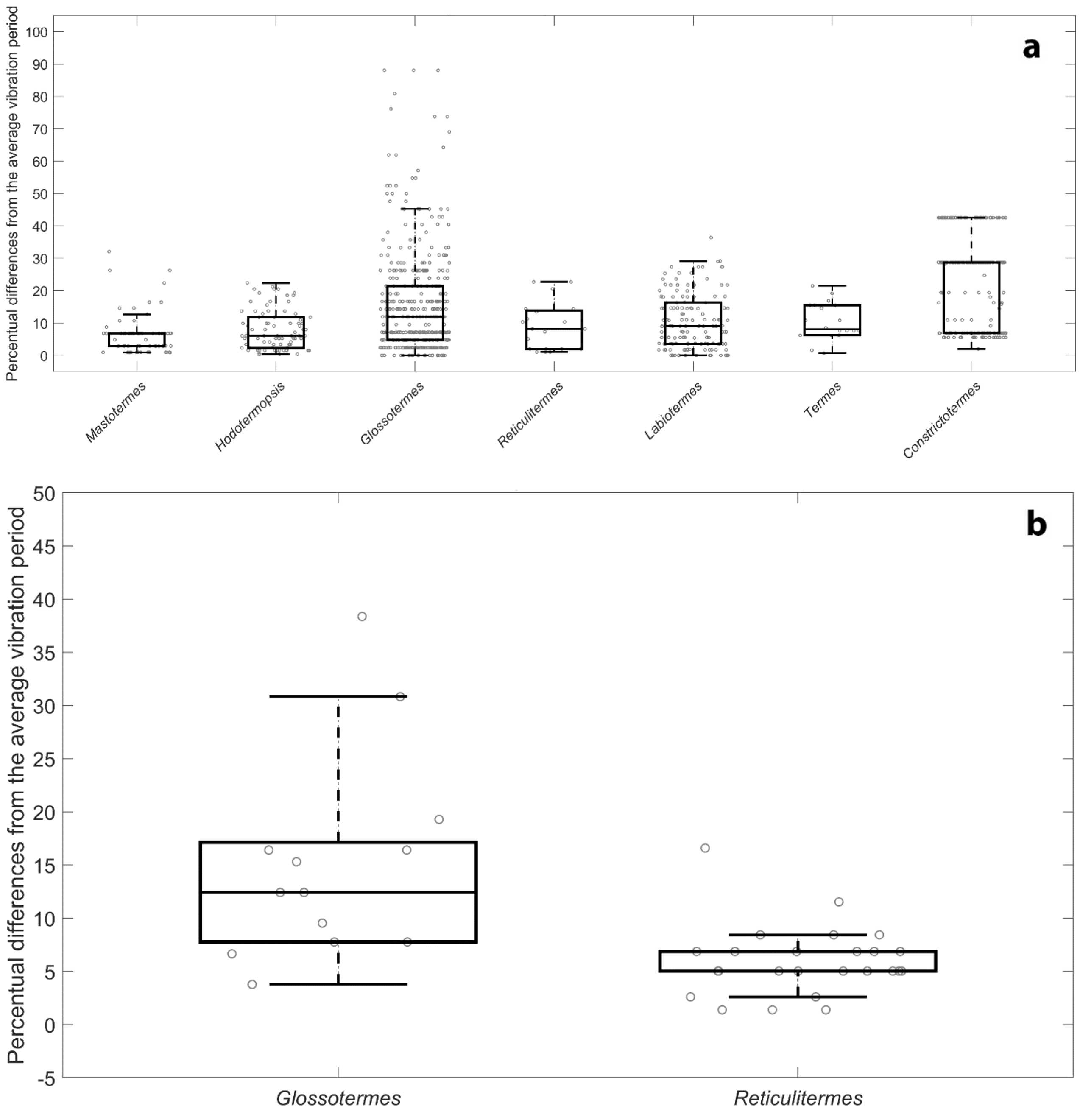

Fig. S5: Multiple Correspondence Analysis (MCA) analysis showing the ecological variables with the strongest effects upon the vibroacoustic communication. Constraining variables of the MCA analyses were counted for the strongest effect in the three first axes and they presented 6 variables (family, food source, nest material, foraging area, chemical alarm, and food hardness) which showed distribution for the most significant axes. See Dataset S1 for characters' legends.

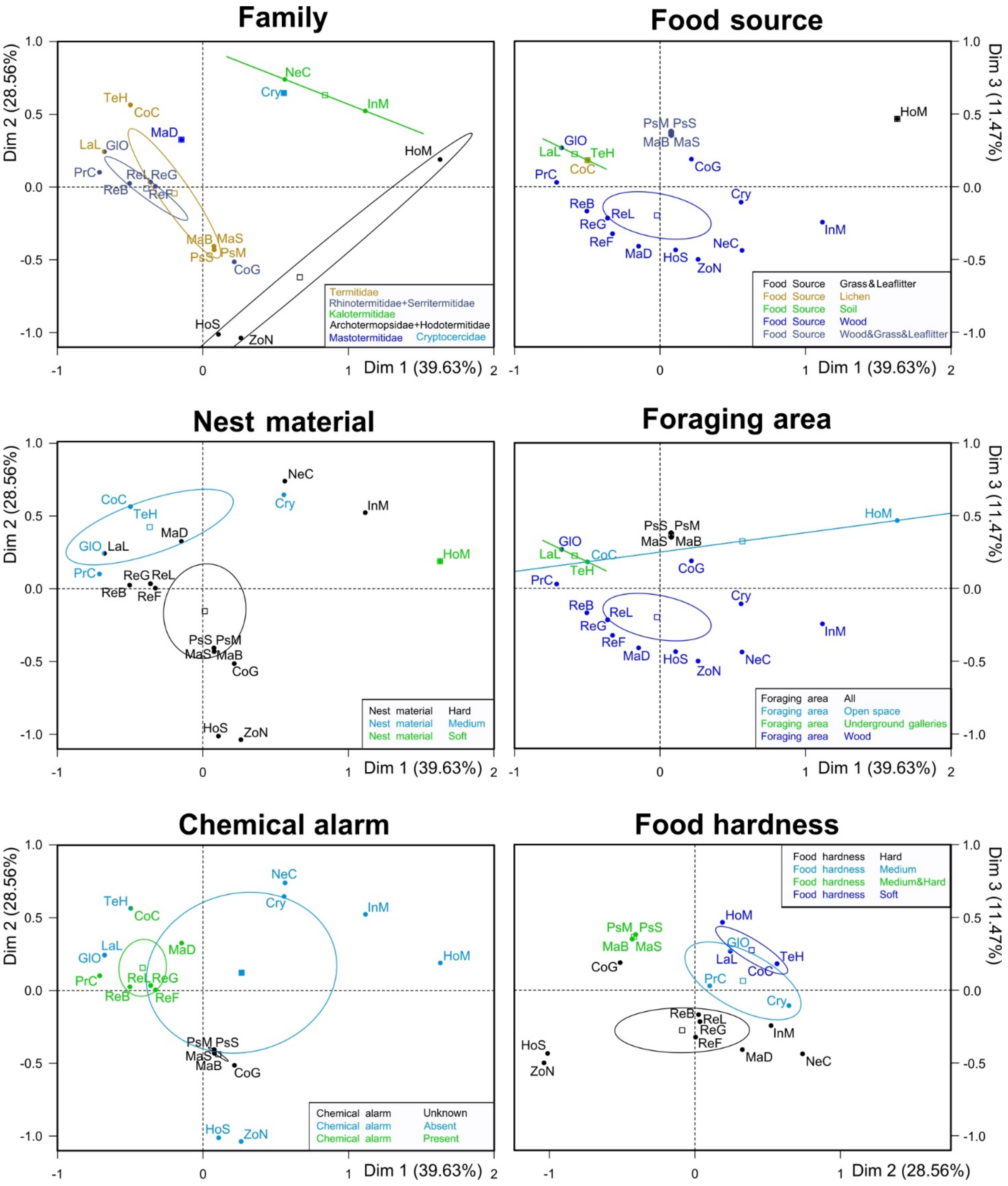

Fig. S6: Workflow of vibroacoustic recording. Detailed explanation of data acquisition for the vibroacoustic experiments.

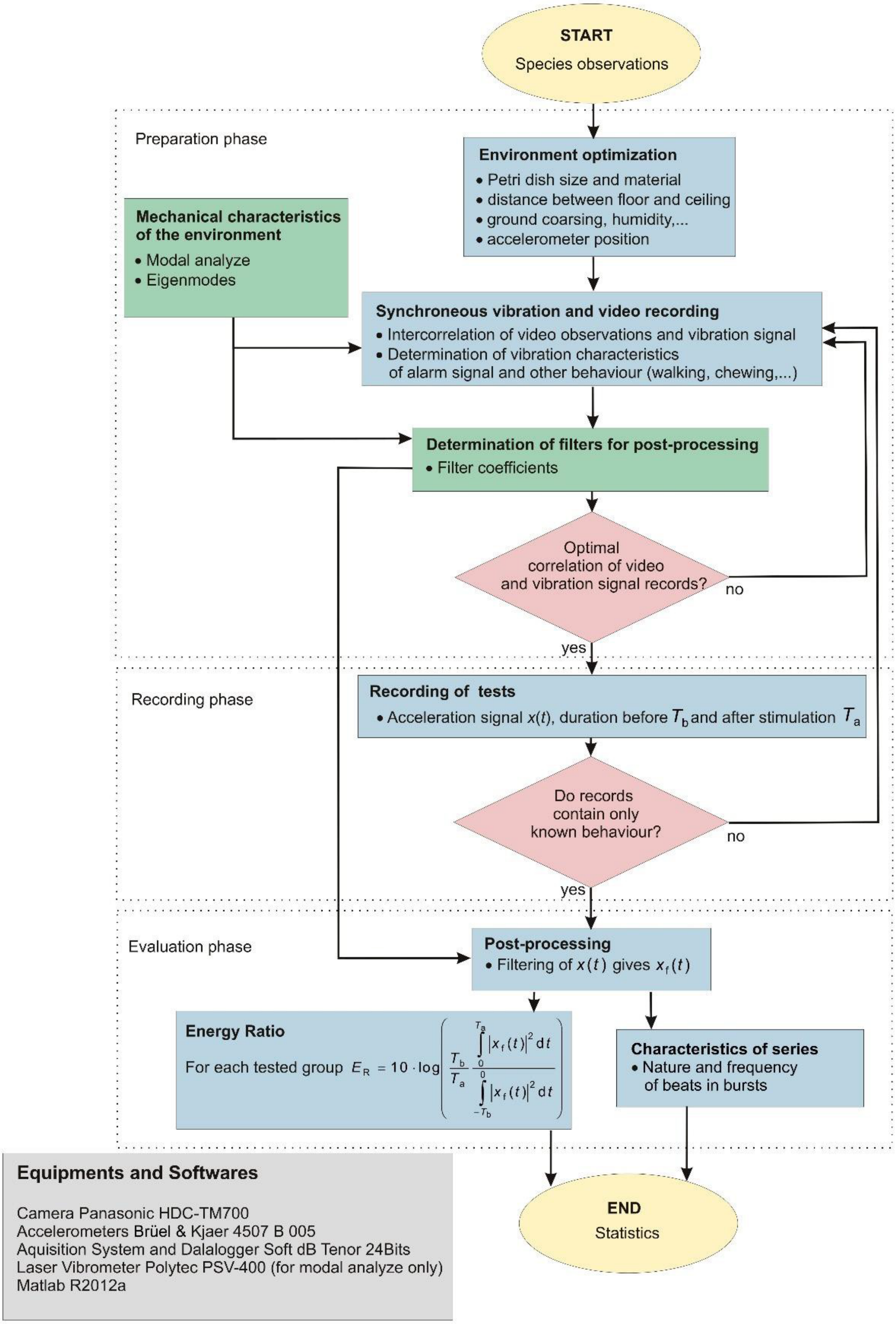

Table S1: Normalized speed-of-motion (mm/s ± SE) of nymphs in the wood roach *Cryptocercus*, and of workers and soldier within termite groups in natural caste ratio (according to Haverty (2) or to our unpublished observations; for details see Dataset S1) in Petri dish stimulated with light flash (light), air current (air), one crushed worker head (CWH) or one crushed soldier head (CSH). The locomotion speed is given as an average scored from two nymphs in the wood roach, or from two workers and two soldiers in termites per experiment. The changes in locomotion speed for workers and soldiers, and the respective P-values for all comparisons between stimulations and controls are presented for short-term (1 min) and long- term (6 min) response to the stimuli. Negative values indicate that termites were more active prior to the stimulation. Kruskal-Wallis and post-hoc two-by-two permutation tests results are given for workers and soldiers independently. The value of Kruskal-Wallis test is given (H) and *P'*-values for all tests are given for P < 0.05 at the last line for each species. "NS" indicates non-significant results. "NA" indicates not applicable. "-" indicates that the overall comparison (Kruskal-Wallis test) was not significant in one given species.

	Short-term response to disturbance								Long-term response to disturbance							
	Workers				Soldiers				Workers				Soldiers			
	Light	Air	CWH	CSH	Light	Air	CWH	CSH	Light	Air	CWH	CSH	Light	Air	CWH	CSH
Cryptocercus	0.02±0.13	2.08±0.13	0.71±0.16	NA	NA				-0.22±0.19	0.78±0.16	0.83±0.13	NA	NA			
		H = 19.62; P<0.001								H = 11.80; P=0.003						
	NS	<0.001	*0.015	NA					NS	0.006	*0.002	NA				
Mastotermes	3.35±4.77	5.59±3.83	1.03±4.10	4.40±7.54	0.56±2.48	3.55±6.14	2.14±5.95	3.18±3.86	3.85±3.20	7.78±2.57	7.15±5.18	11.25±7.38	-0.72±3.63	1.16±3.10	5.08±4.09	6.25±7.01
		H = 18.1; P=0.0018				H = 9.49; P=0.087				H = 36.76; P<0.001				H = 26.58; P<0.001		
	NS	0.002	NS	NS	-	-	-	-	<0.001	0.0012	0.028	0.04	NS	NS	0.004	0.02
Hodotermopsis	0.04±0.61	2.92±1.06	1.13±1.17	0.15±1.08	-0.36±1.15	1.36±1.12	0.13±0.47	0.17±0.74	0.07±0.87	0.26±0.53	0.69±0.76	0.09±1.26	-0.59±1.21	0.28±0.97	0.40±0.89	0.53±1.09
		H = 29.16; P< 0.001				H = 16.36; P= 0.0028				H = 5.048; P= 0.172				H = 3.274; P= 0.355		
	NS	<0.001	NS	NS	NS	NS	NS	NS	-	-	-	-	-	-	-	-
Hodotermes	0.24±0.98	2.56±4.62	1.71±2.75	0.05±3.00	0.97±2.03	1.92±5.31	0.05±1.23	0.83±1.25	0.85±2.78	2.32±5.66	7.66±15.23	0.07±13.21	2.67±4.22	4.39±11.33	0.83±4.28	4.71±3.84
		H = 8.454; P=0.131				H = 7.457; P=0.1922				H = 5.091; P= 0.282				H = 8.253; P= 0.086		
	-	-	-	-	-	-	-	-	-	-	-	-	-	-	-	-
Neotermes	3.24±2.44	7.64±4.31	-0.1±0.65	-0.11±1.32	2.75±2.54	5.99±2.82	0.18±1.06	0.15±1.11	0.76±1.26	0.39±1.05	2.67±1.21	1.86±1.04	0.22±0.68	-0.19±1.19	0.18±1.06	-0.02±1.22
		H = 44.54; P< 0.001				H = 39.14; P< 0.001				H = 22.51; P< 0.001				H = 18.77; P< 0.001		
	<0.001	<0.001	NS	NS	<0.001	<0.001	NS	NS	NS	<0.001	NS	NS	NS	<0.001	NS	NS
Glossotermes	0.35±0.59	1.89±1.17	0.63±0.69	1.42±1.63	-0.22±0.59	0.78±0.68	0.2±0.7	0.5±0.5	0.63±0.61	0.66±0.95	0.47±0.6	1.24±1.41	-0.02±0.73	0.32±0.7	0.16±0.69	0.36±0.60
		H = 31;03; P< 0.001				H = 27.21; P< 0.001				H = 19.14; P< 0.001				H = 3.3; P= 0.668		
	NS	<0.001	0.039	0.02	NS	<0.001	0.08	0.001	<0.001	<0.001	NS	NS	-	-	-	-
Reticulitermes	3.26±1.81	9.68±4.07	1.34±1.32	4.13±1.95	2.02±1.88	4.98±2.47	1.07±1.68	3.06±3.51	2.07±1.39	5.32±2.69	1.30±0.85	4.20±1.39	1.67±0.99	1.82±2.35	0.46±0.98	2.63±2.38
		H = 46; P< 0.001				H = 39.61; P< 0.001				H = 46.01; P< 0.001				H = 27.24; P< 0.001		
	< 0.001	< 0.001	NS	< 0.001	< 0.001	< 0.001	0.002	< 0.001	< 0.001	< 0.001	NS	< 0.001	< 0.001	0.035	NS	0.003
Labiotermes	0.53±0.81	1.58±1.15	0.54±0.87	1.02±1.08	0.31±1.23	1.41±1.32	0.49±0.9	0.81±0.87	1.04±0.73	1.10±0.76	0.53±0.69	1.02±0.88	-0.16±0.74	0.41±0.65	0.32±1.06	0.57±0.76
		H = 9.07; P= 0.097				H = 14.31; P= 0.01				H = 32.63; P= 0.447				H = 10.26; P= 0.065		
	-	-	-	-	NS	0.03	NS	NS	-	-	-	-	NS	NS	NS	NS
Termes	0.99±1.07	1.91±1.99	0.26±0.59	0.48±1.05	-0.4±0.93	2.17±2.58	0.24±1.23	0.14±1.36	1.14±0.30	-0.66±0.97	1.07±0.79	0.96±1.32	0.65±0.92	0.39±0.99	0.61±1.01	0.08±1.23
		H = 13.39; P= 0.018				H = 17.76; P = 0.002				H = 11.35; P= 0.039				H = 14.32; P= 0.009		
	0.081	NS	NS	NS	< 0.001	NS	NS	NS	NS	NS	NS	NS	NS	NS	NS	NS

Table S2: Overview of responses (running and/or vibrating) of workers and soldiers of different termite species after exposure to crushed soldier head (CSH – alarm pheromone simulation) in the experimental conditions of Table S1. Increased locomotion activity (running) or vibration activity indicates behavioural response to alarm pheromone.

	Soldier response		Worker response		
	**Running**	**Vibrating**	**Running**	**Vibrating**	**Reference**
Mastotermes darwiniensis	NO	YES	YES	YES	Delattre et al. 2015
Reticulitermes flavipes	NO	YES	YES	YES	Delattre et al.2019
Constrictotermes cyphegaster	YES	YES	YES	YES	Cristaldo et al. 2015
Prorhinotermes canalifrons	YES	YES	YES	YES	Šobotník et al. 2008
Reticulitermes grassei	YES	Unknown	YES	Unknown	Reinhard & Clément 2002
Reticulitermes lucifugus	YES	Unknown	YES	Unknown	Reinhard & Clément 2002
Reticulitermes banyulensis	YES	Unknown	YES	Unknown	Reinhard & Clément 2002

Table S3: Long-term differences in vibroacoustic alarm signaling after air blow expressed as normalized activity prior vs. after disturbance. Termites usually responded by increased vibroacoustic activity after the disturbance. The exception of *Hodotermes* is due the fact that according our knowledge it misses any alarm communication.

	N	df	E_R	t	p-value
Mastotermes	8	7	3.43±3.5	-2.771	0.028
Hodotermopsis	4	3	6.42±1.27	-10.116	0.002
Neotermes	4	3	8.2±2.32	-7.073	0.006
Glossotermes	4	3	-0.2±1.54	0.26	NS
Reticulitermes	4	3	5.6±1.9	-5.888	0.01
Labiotermes	4	3	3.75±0.28	-26.406	<0.001
Termes	7	6	1.83±0.79	-6.09	<0.001

Table S4: Identity of the compounds (detected from glandular extracts or SPME collections) released from labial glands or the frontal gland in the termite species studied. Some of the compounds are used as alarm pheromones (CHC = cuticular hydrocarbons; Ø = no compound detected; N.A. = not available).

	Extract		SPME		Reference
	Workers	Soldiers	Workers	Soldiers	
Mastotermes darwiniensis	CHC	CHC + *p*-benzoquinone	Ø	*p*-benzoquinone	Moore 1968, Delattre et al. 2015
Hodotermopsis sjostedti	CHC	CHC	Ø	CHC	Current study
Hodotermes mossambicus	CHC	CHC	Ø	Ø	Current study
Neotermes cubanus	CHC	CHC	Ø	Ø	Current study
Glossotermes oculatus	Ø	CHC	Ø	Ø	Current study
Reticulitermes flavipes	CHC	3-carene or α-pinene /β-pinene/ limonene	Ø	α-pinene or 3-carene/ limonene or sylvestrene / β-pinene/unknown	Delattre et al. 2019
Labiotermes labralis	CHC	Ø	Ø	methyl ester octanoid acid	Current study
Termes hospes	CHC	Terpene	Ø	C9-C17 HC	Current study
Constrictotermes cyphergaster	CHC	CHC, monoterpenes, sesquiterpenes, and diterpenes	N.A.	N.A.	Cristaldo et al. 2015

References

1. Abe, T. “Evolution of life types in termites” in *Evolution and Coadaptation in Biotic Communities*, S. Kawano, J. H. Connell, and T. Hidaka, Eds. (1987) pp. 125–148.

2. Haverty, M. I. The proportion of soldiers in termite colonies: a list and a bibliography. *Sociobiology* 2, 199–216 (1977).
