# Supplementary material for: Alarm communication predates eusociality in termites
Source: Commun Biol. 2023 Jan 21;6:83. doi: 10.1038/s42003-023-04438-5 (PMC9867704; doi:10.1038/s42003-023-04438-5)
Supplement: Supplementary file 3 — Description of Additional Supplementary Files [file 42003_2023_4438_MOESM3_ESM.pdf]

Description of Additional Supplementary Files

File name: Supplementary Data 1
Description: List of characters (from X1 to X33) from the wood roach Cryptocercus punctulatus and from termites. Information about alarm characters is retrieved from literature when indicated or from our experiments when no literature reference is provided (in that case, the country of collection of the species is given). All characters are used in the analysis (except X1-X4 which have been excluded from Multiple Correspondence Analysis because they resulted from other characteristics). "?" means that no information is available or the character is inapplicable. Tables and figures with species codes refer to the ones of this table. The caste ratio of workers and soldiers used in the experimental groups is indicated for the species studied in the behavioural experiments. Life type refers to the classification of Abe (1), i.e. one-piece lifetype for termites that feed on wood in which they also nest, intermediate life-type for termites which nest in wood but can colonize new food sources through underground galleries, and separate life-type for species in which workers forage outside the nest.

File name: Supplementary Data 2
Description: Figure 4 Raw data.

File name: Supplementary Data 3
Description: Figure 5 Raw data.

File name: Supplementary Data 4
Description: Figure S4a Raw data.

File name: Supplementary Data 5
Description: Figure S4b Raw data.

File name: Movie S1
Description: Experimental set-up to record in an anechoic room the tremulations (vibroacoustic behaviour) of individuals of the wood roach Cryptocercus punctulatus and the termites Mastotermes darwiniensis, Hodotermopsis sjostedti, Neotermes cubanus, Glossotermes oculatus, Reticulitermes flavipes, Labiotermes labralis, Termes hospes, and Constrictotermes cyphergaster. The circles show one specific individual whose vibroacoustic communication displayed on the following graph.
