# Supplementary material for: Alarm communication predates eusociality in termites
Source: Commun Biol. 2023 Jan 21;6:83. doi: 10.1038/s42003-023-04438-5 (PMC9867704; doi:10.1038/s42003-023-04438-5)
Supplement: Supplementary file 11 — Reporting Summary [file 42003_2023_4438_MOESM11_ESM.pdf]

nature portfolio

Corresponding author(s): Jan Šobotník and Michael S. Engel

Last updated by author(s): Oct 16, 2022

Reporting Summary

Nature Portfolio wishes to improve the reproducibility of the work that we publish. This form provides structure for consistency and transparency in reporting. For further information on Nature Portfolio policies, see our Editorial Policies and the Editorial Policy Checklist.

Statistics

For all statistical analyses, confirm that the following items are present in the figure legend, table legend, main text, or Methods section.

n/a	Confirmed	
☐	☒	The exact sample size (*n*) for each experimental group/condition, given as a discrete number and unit of measurement
☐	☒	A statement on whether measurements were taken from distinct samples or whether the same sample was measured repeatedly
☐	☒	The statistical test(s) used AND whether they are one- or two-sided<br>*Only common tests should be described solely by name; describe more complex techniques in the Methods section.*
☐	☒	A description of all covariates tested
☐	☒	A description of any assumptions or corrections, such as tests of normality and adjustment for multiple comparisons
☐	☒	A full description of the statistical parameters including central tendency (e.g. means) or other basic estimates (e.g. regression coefficient) AND variation (e.g. standard deviation) or associated estimates of uncertainty (e.g. confidence intervals)
☐	☒	For null hypothesis testing, the test statistic (e.g. *F*, *t*, *r*) with confidence intervals, effect sizes, degrees of freedom and *P* value noted<br>*Give P values as exact values whenever suitable.*
☒	☐	For Bayesian analysis, information on the choice of priors and Markov chain Monte Carlo settings
☐	☒	For hierarchical and complex designs, identification of the appropriate level for tests and full reporting of outcomes
☐	☒	Estimates of effect sizes (e.g. Cohen's *d*, Pearson's *r*), indicating how they were calculated

Our web collection on statistics for biologists contains articles on many of the points above.

Software and code

Policy information about availability of computer code

Data collection	MouseTracker (self-made software tracking position of randomly selected termites), Audacity, GC Agilent Software
Data analysis	Mesquite, StatXact, SigmaPlot, R software

For manuscripts utilizing custom algorithms or software that are central to the research but not yet described in published literature, software must be made available to editors and reviewers. We strongly encourage code deposition in a community repository (e.g. GitHub). See the Nature Portfolio guidelines for submitting code & software for further information.

Data

Policy information about availability of data

All manuscripts must include a data availability statement. This statement should provide the following information, where applicable:

- Accession codes, unique identifiers, or web links for publicly available datasets
- A description of any restrictions on data availability
- For clinical datasets or third party data, please ensure that the statement adheres to our policy

Most of the datasets analysed in the current study are included in supplementary on-line materials.

Human research participants

Policy information about studies involving human research participants and Sex and Gender in Research.

Reporting on sex and gender	N/A
Population characteristics	N/A
Recruitment	N/A
Ethics oversight	N/A

Note that full information on the approval of the study protocol must also be provided in the manuscript.

Field-specific reporting

Please select the one below that is the best fit for your research. If you are not sure, read the appropriate sections before making your selection.

☐ Life sciences ☐ Behavioural & social sciences ☒ Ecological, evolutionary & environmental sciences

For a reference copy of the document with all sections, see nature.com/documents/nr-reporting-summary-flat.pdf

Ecological, evolutionary & environmental sciences study design

All studies must disclose on these points even when the disclosure is negative.

Study description	We studied evolution of alarm communication in termites. We believe the manuscript and supplementary materials describe the study design into necessary details.
Research sample	We selected 9 species of termites spread across the tree of life plus a wood roach, member of sister group to all termites. Groups of every species close to natural caste ratio were recorded in an anechoic room, and confronted to three potentially endangering stimuli. All species were studied de novo, however, for Constrictotermes cyphergaster, we used data previously recorded in the similar manner by part of the team (published in Cristaldo et al. 2015) and re-analysed them in order to achieve a broader goal.
Sampling strategy	The particular samples were prepared based on our previous experiences, to represent a viable functional units.
Data collection	Author Contributions: DSD, formulation of research strategy, supervisor of behavioral part, writing the MS; VJ, vibroacoustic recording and signal processing, data analysis, writing the MS; PS, statistical and ecological modelling; OD behavioral tests with termites, compiling the literature sources; TC, writing the MS, data analysis; OB, behavioral tests with Cryptocercus, evaluation of behavior; JC, chemical analyses; DS, behavioral results evaluation; JS, preparation of experiments, behavioral results evaluation; MB and OJ, evaluation of vibroacoustic data; MSE and TB, results presentation, text edits; JŠ, formulation of research strategy, funding, coordination of all activities, writing the MS.
Timing and spatial scale	The work on particular species started in 2015, and were processed on by one according to material availability till 2018. Subsequently, the data were analysed and statistically evaluated. All practical works took place in anechoic room at Czech Technical University in Prague, Faculty of Electrical Engineering.
Data exclusions	No data were excluded.
Reproducibility	We present all data allowing others to repeat our work.
Randomization	The material was not intentionaly randomised, however, the groups were created de novo from the stock colonies. Used material was never reused again.
Blinding	This is a complex story conducted by a broad team, and each member was responsible for different part of the data acquisition and analysis. We have not applied any a priori blinding strategy.

Did the study involve field work? ☐ Yes ☒ No

Reporting for specific materials, systems and methods

We require information from authors about some types of materials, experimental systems and methods used in many studies. Here, indicate whether each material, system or method listed is relevant to your study. If you are not sure if a list item applies to your research, read the appropriate section before selecting a response.

Materials & experimental systems

n/a	Involved in the study
☒	☐ Antibodies
☒	☐ Eukaryotic cell lines
☒	☐ Palaeontology and archaeology
☐	☒ Animals and other organisms
☒	☐ Clinical data
☒	☐ Dual use research of concern

Methods

n/a	Involved in the study
☒	☐ ChIP-seq
☒	☐ Flow cytometry
☒	☐ MRI-based neuroimaging

Animals and other research organisms

Policy information about studies involving animals; ARRIVE guidelines recommended for reporting animal research, and Sex and Gender in Research

Laboratory animals	We mostly worked with parts of laboratory colonies of termites.
Wild animals	Part of material originated from the field. For this purpose, we transported fractions of termite nests with sufficient population inside, based on legal procedures, as specified in the manuscript now.
Reporting on sex	N/A
Field-collected samples	All material was, except for the recording period, help in laboratory breeds at constant darkness and temperature +27°C .
Ethics oversight	The termite material was maintained at Czech University of Life Sciences Prague, and the experimentation took place at Czech Technical University in Prague. Both Universities had no objections against conducting this kind of research.

Note that full information on the approval of the study protocol must also be provided in the manuscript.
